# Modern Advances in CARs Therapy and Creating a New Approach to Future Treatment

**DOI:** 10.3390/ijms232315006

**Published:** 2022-11-30

**Authors:** Karol Sadowski, Wioletta Olejarz, Grzegorz Basak

**Affiliations:** 1 Department of Hematology, Transplantation and Internal Medicine, Medical University of Warsaw, 02-091 Warsaw, Poland; 2 Department of Biochemistry and Pharmacogenomics, Faculty of Pharmacy, Medical University of Warsaw, 02-091 Warsaw, Poland; 3 Centre for Preclinical Research, Medical University of Warsaw, 02-091 Warsaw, Poland

**Keywords:** immunotherapy, CARs therapy, checkpoint inhibitors, exosomes

## Abstract

Genetically engineered T and NK cells expressing a chimeric antigen receptor (CAR) are promising cytotoxic cells for the treatment of hematological malignancies and solid tumors. Despite the successful therapies using CAR-T cells, they have some disadvantages, such as cytokine release syndrome (CRS), neurotoxicity, or graft-versus-host-disease (GVHD). CAR-NK cells have lack or minimal cytokine release syndrome and neurotoxicity, but also multiple mechanisms of cytotoxic activity. NK cells are suitable for developing an “off the shelf” therapeutic product that causes little or no graft versus host disease (GvHD), but they are more sensitive to apoptosis and have low levels of gene expression compared to CAR-T cells. To avoid these adverse effects, further developments need to be considered to enhance the effectiveness of adoptive cellular immunotherapy. A promising approach to enhance the effectiveness of adoptive cellular immunotherapy is overcoming terminal differentiation or senescence and exhaustion of T cells. In this case, EVs derived from immune cells in combination therapy with drugs may be considered in the treatment of cancer patients, especially effector T and NK cells-derived exosomes with the cytotoxic activity of their original cells.

## 1. Introduction

CARs therapy is a type of immunotherapy in which patient-derived T cells or NK cells are genetically engineered ex vivo to kill cancer cells and are subsequently delivered back into the patient [1]. These cells express Chimeric Antigen Receptors (CARs), which bind specific antigens on the surface of cancer cells and trigger a cytotoxic response [2]. In 1993, Zelig Eshhar developed first-generation CARs and provided a T-cell receptor (TCR)-like “signal 1” via CD3ζ or FcεRIγ [3]. Genetically engineered T and NK cells expressing a chimeric antigen receptor are promising cytotoxic cells for the treatment of hematological malignancies and solid tumors [4]. CAR engineering can redirect the specificity of immune effector cells by inducing rapid and durable clinical responses [5]. CARs contain antigen-recognition regions as a single-chain variable fragment (scFv) or a binding receptor/ligand in the extracellular domains. T cells have TCR CD3ζ chain that provides “signal 1” and one or more domains from costimulatory receptors, such as CD28, OX40 (CD134), and/or 4-1BB (CD137), to provide “signal 2” [6]. Alongside natural cytotoxic activity against tumor cells, CAR-NK cells can also be activated through CAR-independent mechanisms, such as NCRs, NKG2D, costimulatory receptor DNAM-1 (CD226), certain activating KIRs, and through CD16-mediated ADCC [7,8]. Acute and chronic B-cell leukemia and B-cell non-Hodgkin lymphomas (NHLs) with anti-CD19 CAR-T cells are adoptive T-cell therapies approved by the Food and Drug Administration (FDA). This approach is associated with an overall response rate of 50–90% in patients with B-cell malignancies refractory to standard therapies [9,10]. In this text, we focus on the difference between CAR-T and CAR-NK cells and their advantages/disadvantages, which are important to choose the right treatment approach.

## 2. CARs

CARs are engineered synthetic receptors that mainly target lymphocytes against cells expressing a specific target antigen. CAR binding to target antigens on the cell surface is independent of the MHC receptor. That results in vigorous T cell activation and effective anti-tumor responses [11,12]. One of the ways to improve functionality is to look for new and more efficient components in constructing CAR-T cells. Below we have described each element and possible aspects of their modification.

**Figure 1:**
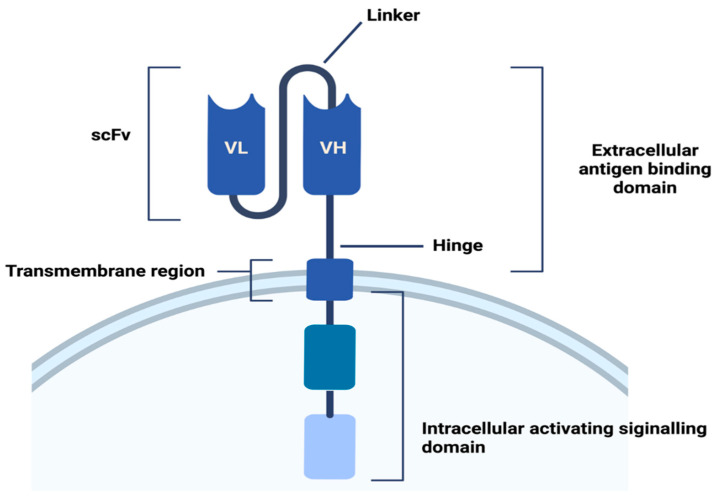
Structure of CAR receptor [16].

The extracellular antigen binding domain binds to specific antigens of tumor cells. Thus, it induces CAR signaling and activates T cells [13]. A proper function is ensured by moderate antigen-binding affinity; low-affinity results in activation-induced death of the CAR-expressing T cell and triggers toxicities. The extracellular antigen binding domain mostly takes the form of single-chain variable fragments [14,15]. The hinge is an extracellular structure that extends the binding units from the transmembrane domain. The hinge by flexibility allows the steric hindrance to be overcome and contributes to the antigen-binding domain reaching the epitope. The transmembrane region attaches the extracellular components to the membrane [16] (Figure 1).

**Figure 2:**
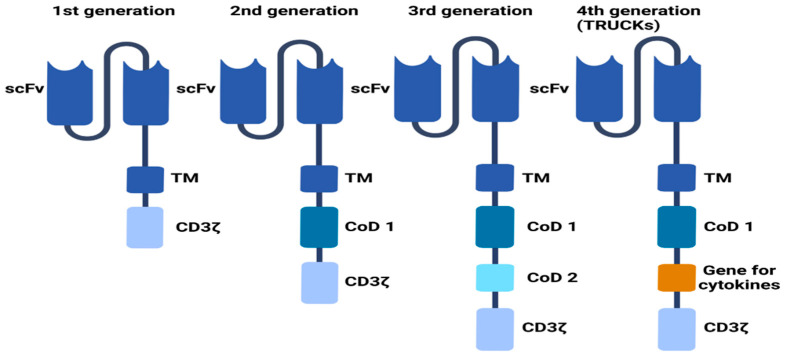
Generations of CAR-T cells. 1st generation of CAR-T cells is based on the presence of the intracellular CD3ζ domain. 2nd generation incorporates an additional costimulatory domain (CoD), mostly CD28. 3rd generation contains two costimulatory domains, e.g., CD28, ICOS, 4-1BB, or OX40. These domains activate multiple signaling pathways. This provides a more effective approach to aiming to destroy cancer cells. 4th generation (also called TRUCKs) is based on the second generation with an additional gene responsible for cytokine expression in the cell microenvironment [11,21,22,23,24,25,26,27].

More evidence suggests that the transmembrane domain could modulate the CAR-T cell function [17,18]. Most are derived from natural proteins, such as CD3ζ, CD4, CD8α, or CD28. This element of CARs is frequently changed for the needs of extracellular components or intracellular signaling domains. It was shown that both the transmembrane domain and hinge region appear to modulate CAR-T cell cytokine production and activation-induced cell death (AICD). CAR-T cells with CD8α transmembrane and hinge domains release decreased amounts of TNF and IFNγ and have a reduced sensitivity to AICD relative to CARs with these CD28-derived domains [19]. The internal part of CARs is the intracellular activating signaling domain, which mediates the activation and cytotoxicity of CAR cells. The first domain contains the FcRγ signaling domain or a CD3ζ that activates CAR [2,20]. Depending on the domains structure, individual CARs have different properties. Based on their combinations and structure, the CARs could be divided to four generations [11,21,22,23,24,25,26,27] (Figure 2).

## 3. CAR-T Cytotoxic Mechanism

CAR-T cells are genetically engineered by inserting genes coding receptors capable of recognizing specific neoplastic cells. They can recognize antigens regardless of MHC presentation. However, they are limited to recognizing structures expressed at the surface [28,29]. The functionality of CAR-T cells conforms to several mechanisms: formation of the immunological synapse (IS), exocytosis of receptor-mediated apoptosis via the expression of Fas ligand or TRAIL, cytolytic granules, and cytokine production [30,31]. Another classification subdivides mechanisms into slow-acting (TNF ligands family) and fast-acting (degranulation) killing mechanisms [30]. Each of these stages/elements can be subject to modification, which can contribute to increasing or decreasing the efficiency the therapy itself. The first of them is IS, which in CAR-T is slightly different. The diameter of CAR-T IS is smaller, which correlates with a faster CAR-T cell detachment [31]. CAR-T cells lyse the antigen-positive cancer cells mainly by the granzyme and perforin. Thus, degranulation is crucial for rapid and specific CAR-T cell-induced target cell lysis. The granules with their cytolytic payload are released in the central part of the synaptic cleft—cSMAC [32]. Released perforin induces pore formation in the cell membrane, forming a way for granzymes. These two mechanisms lead the cell to the induction of a caspase-dependent and -independent apoptosis [33,34]. Therefore, cytolytic degranulation is assumed to be the most important mechanism of cell killing by CAR-T cells [35,36,37]. The mechanisms of cytotoxicity are analyzed in the hope of enhancing particular points of grip.

Fas and Fas Ligand pathways classically involved in immune cell homeostasis in non-pathogenic situations also can activate the mechanism to mediate neoplastic cells. The antigen-negative cancer cells can be targeted via FAS and Fas Ligand axis, independent of presenting death receptors by the cancer cell. It is estimated that the functions of this pathway may be pivotal in the heterogeneous environment of the tumor [38,39,40].

The cytokines enhance both these mechanisms. Modulating features of cytokine enables the response to be highly specific and more effective in cases of phenotypic diversity of cancer cells. Their secretion by CAR-T cells mediates tumor lysis via upregulating IFN-gamma on stromal cells [41]. This leads to immune cell modulations, such as the polarization of macrophages to the antitumoral M1 phenotype [42].

**Figure 3:**
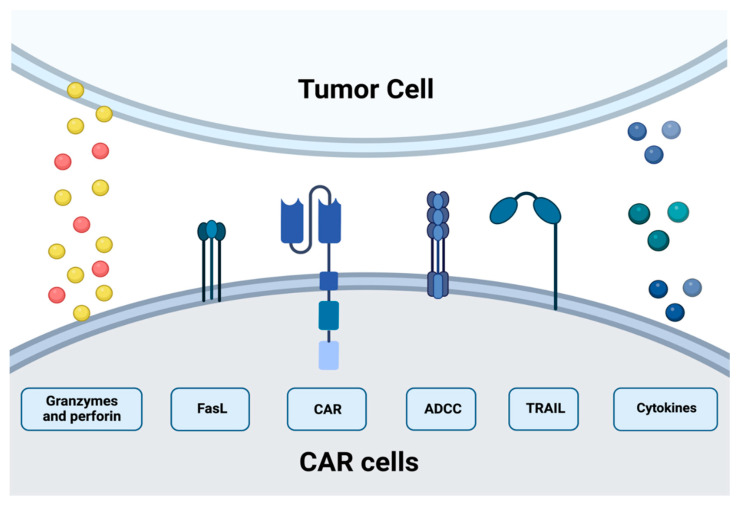
The cytotoxic mechanism of CARs cells.

The ability to kill multiple target cells sequentially has been demonstrated for natural cytotoxic lymphocytes, such as NK and Tc. The rapid destruction by the relatively low number of effector cells has also been confirmed in CAR-T cells [36]. Interestingly, some studies show superiority over T cells—shorter time of synapse formation or stronger signal strength in IS. During engagement, quantified granzyme and perforin release were comparable but later in the case of T cells. Despite that, the serial killing events were equal for both types of cells [31,36] (Figure 3).

## 4. CAR-T Therapy

CAR-T cell therapy is the first gene therapy approved by U.S. Food and Drug Administration [43]. In treating refractory/relapsed acute lymphoblastic leukemia in children and young adults as well as relapsed/refractory diffuse large B-cell lymphoma (DLBLC) tisagenlecleucel (Kymriah™) is used, while in the treatment of relapsed/refractory high-grade B-cell lymphoma and primary mediastinal B-cell lymphoma axicabtagene ciloleucel (Yescarta™) is used. This therapy, by genetically engineered autologous T cells as “living drugs” targeting CD19, was approved in Europe and prepared under the auspices of the European Society of Blood and Marrow Transplantation (EBMT) and the Joint Accreditation Committee of ISCT and EBMT (JACIE) [44]. Brexucabtagene autoleucel (Tecartus™) was approved by the FDA in highly refractory patients with mantle cell lymphoma (MCL) [45], and in adults with relapsed and refractory B-cell acute lymphoblastic leukemia (ALL) [46]. For the treatment of B cells, non-Hodgkin lymphoma (NHL) lisocabtagene maraleucel (Breyanzi™) has been approved [47].

Importantly, the most promising approach against refractory/relapsed multiple myeloma (MM), which is unresponsive to any other currently known treatment, is the combination of CAR-T cells with other drugs, such as monoclonal antibodies, proteasome inhibitors, or new immunomodulatory drugs [48]. Data from clinical trials have demonstrated that patients with relapsed and/or refractory MM can achieve objective responses in short-term safety and efficacy by applying anti-BCMA (B cell maturation antigen) CAR-T cells [49] (Table 1).

## 5. Limitations of CAR-T Cells Therapy

The increasing knowledge about CAR-T cell therapy tells us not only about its advantages but also about its limitations. The main problems of CAR-T cells therapy are cytokine release syndrome (CRS), neurotoxicity, or graft-versus-host-disease (GVHD) [50]. Different cytokine secretion profiles in CAR-T cells lead to various symptoms, such as high fever, sinus tachycardia, hypotension, hypoxia, depressed cardiac function, and other organ dysfunction [51]. Additionally, dysfunction of CAR-T cells due to exhaustion and senescence are a key hurdle for the success of this therapy [52]. Growing evidence has confirmed that exhausted T cells cause poor responsiveness to immune-checkpoint-blockade therapies and dampen effector immunity [53,54]. Subsequent studies and guidelines on production or storage methods are helping to reduce them or rule them out altogether. A comparison between autologous and allogeneic cells used in CAR-T therapy is presented in the Table 2 [55,56,57,58].

## 6. CAR-NK Cytotoxic Mechanism

NK cells are a type of cytotoxic lymphocyte crucial in the innate immune system, and they constitute 5–15% of human peripheral blood leukocytes. Their distinctive feature is an expression of CD56 without co-expression of CD3 and T cell receptors (TCR) [59]. Their ability for cytotoxicity depends on variable mechanisms is described in Table 3 [38]. For instance, there are a series of activating receptors such as TNF-related apoptosis-inducing ligands (TRAILs), costimulatory receptors (e.g., CD244, CD137), and the well-established subsets (e.g., FcgRIIIa, FasL, NKG2D, NKp44, NKp46), which are capable of provoking cytolytic programs via intra-cytoplasmic ITAMs (e.g., 2B4, 41BB) [60,61,62]. Therefore, these mechanisms also occur in CAR-T cells, but the spectrum of secreted cytokines is different. The activated NK cells usually release IFN-gamma and GM-CSF. [63] However, CAR-T cells secrete: IL-1a, IL-1Ra, IL-2, IL-2Ra, IL-6, TNF-a, MCP- 1, IL-8, IL-10, and IL-15 [64].

## 7. CAR-NK Therapy

The CAR-NK therapy includes two phases. The first part is preparing the body for the modified NK cells through three consecutive days of chemotherapy. After two days, the patient receives a single dose of CAR-NK. The overall success of CAR-NK is combined anti-tumor activity entailed by CAR expression and their natural ability to kill cancer cells. Despite the increasing references to CAR-NK cell-based cancer immunotherapy, most current studies are preclinical. However, the existing studies’ observations favor novel treatment concepts employing CAR-NK cell lines with potent degranulation and selective cytotoxicity in malignancies [77].

## 8. Sources of NK Cells

**Figure 4:**
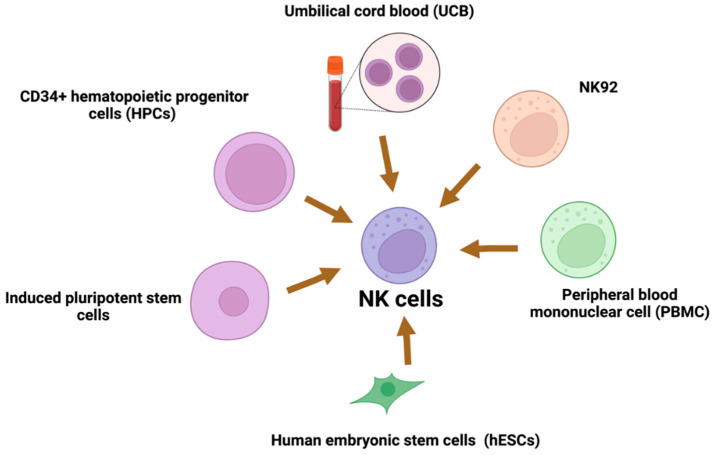
Sources of NK cells.

There are multiple various sources of clinical-grade NK cells. The most popular are described in Figure 4 [78,79]. NK92 cells originated from patients with non-Hodgkin’s lymphoma. Therefore, they require irradiation before the infusion to eliminate the risk of neoplastic transformation and the accompanying chromosomal abnormalities [80]. They have commonly used cell lines in adoptive immunotherapy. They can easily and reproducibly expand from a good manufacturing practice (GMP)-compliant cryopreserved master cell bank. NK92 cells do not express killer immunoglobulin-like receptors (KIRs) or CD16. Thus, they cannot mediate ADCC [81]. They activate cytolytic pathway molecules with perforin and granzyme against neoplastic cells [82].

One of the disadvantages of NK cells is their relatively short persistence in the peripheral blood after infusion. Unmodified NK cells were detected in the circulation for only up to one week after infusion, and NK92 cells for up to 48 h post infusion, which seems to be insufficient for CAR-NK therapy [83,84]. To solve that problem, other human lines have also been evaluated as possible alternatives (e.g., NKL, KHYG-1, YTS, or NKG) [85]. The maturation stage and viability of NK cells depend on the source and preparation; it is implicated in the different anti-tumor effectiveness of produced CAR-NK cells so that similar molecules may have different outcomes.

## 9. Limitations CAR-NK Cells Therapy

The technical problem in CAR-NK therapy is that NK cells are more sensitive to the process of freezing and thawing. This reduces their anti-tumor capacity and survival rate, and limits the immediate use of the therapy and the long-range transport of the modified CAR-NK cells. Still, the capacity of frozen NK cells could be partially recovered by using IL-2 [86,87,88]. In the tumor microenvironment (TME), many immunosuppressive cytokines, e.g., TGFβ, adenosine, and indoleamine 2,3-dioxygenase, decrease the effects of CAR-NK cells [89]. Natural molecules could have a negative impact, and exocrine inhibitory receptors may lead to CAR-NK cell dysfunction. Their operation will be most influenced by immune checkpoint molecules, C- type lectin receptors, and cytokine checkpoints [90]. On the other hand, IL-2, IL-12, and IL-15 are crucial for the proper functioning of NK cells in both innate and adoptive immunotherapy [91].

Although NK cells are considered significant effector cells that mediate early graft versus leukemia (GVL) reaction, they may prevent GVHD by killing recipient antigen-presenting cells (APCs) and cytotoxic T lymphocytes [92]. It was shown that the absence of TCR strongly reduces the risk of GVHD [93,94,95].

Importantly, CAR-NK cells could be more effective against tumor cells than CAR-T cells. This is due to the processes by which CAR-NK cells operate: natural cytotoxicity in case of downregulated expression of targeted tumor antigens, ADCC effect, TNF-related apoptosis-inducing ligand (TRAIL), and FAS/FASL [96,97]. However, the CAR-NK cells have a shorter lifespan of only 1 to 2 weeks in the bloodstream [98]. To ensure their persistence in the donor, additional infusion with cytokines is required.

Significant efforts have been made to enhance CAR-NK cell responses against surface antigens by multiple targeted activations such as CD19, CD20, CD22, CD276, CD138, CS1, HER-2, NKG2D, and GD 2 [99,100,101,102,103]. It was shown that specialized molecules could enhance CAR-NK cells with greater costimulatory specificity (e.g., DAP10, DAP12, and 2B4) than those widely used in CAR-T cells (e.g., CD28 and 4-1BB) [104]. 

Advantages and disadvantages of CAR-T and CAR-NK cells are presented in the Table 4 [58,105].

## 10. Nanobodies Based CARs

Recent years have brought some improvements and new methods to CARs therapy. One of them is use of scFv, which only consists of variable heavy-chain VH connected with light-chain VL by short linker peptide [106,107]. ScFv as a targeting domain could also be used as a component of T-cell-redirecting bispecific antibodies (TRBA), which afford them specificity and high affinity [108]. An even more significant approach is the development of single chain-only antibodies (ScAbs) called nanobodies (Nbs). Nanobodies commonly known as Camelidae-derived single-domain antibodies are the smallest antibody fragments with full antigen-binding capacity. They have properties such as small size, high specificity, strong affinity, excellent stability, and modularity [109].

One application has been identified as a promising innovation of an Nb-CAR-based therapy. In nature, some species of shark and Camelidae produce ScAbs [110,111]. Their small size and manufacturing feasibility means that they are widely applied as the antigen-binding domain of CARs, and their properties allow them to combine into conglomerates with greater bond strength and specificity [112]. Additionally, they retain effective penetration due to their size and stable domain structure [106]. Nbs as an improvement helps to overcome problems of immune reactions against linkers connecting VH and VL domains in normal antibodies [113]. In standard therapy, infusion of CAR-T could initiate/mediate immune reactions against such linkers by producing anti-drug antibodies (ADA) [114,115].

Additionally, most CAR-T cells are from murine sources. Thus, the infusion can act as a trigger for immunization. The formation of human anti-mouse antibodies (HAMAs) can remarkably restrict the functionality of CARs therapy [116,117]. The data indicate that nanobodies are poorly immunogenic and safe for common use [118].

ScFv offers various options for modifying the structure of CARs. The potential cross-pairing of two V domains among two independent scFv molecules results in lower affinity [119]. The nanobodies do not have the limit of affinity loss that is recognized as a possible side effect in scFvs design [120,121]. Therefore, Nbs eliminate the technical problem of inserting large fragments of DNA into retroviral vectors. This standard approach lowers the efficiency of transfection and viral packaging [122,123,124]. Furthermore, they have a longer CDR3 than scFvs, enabling them to reach different epitopes out of range for conventional mAbs [125,126,127].

So far, most developed nanobody-based CAR-T cells targeted receptors or antigens such as VEFGR2, HER2, PSMA, TAG-72 GPC2, CD38, CD33, CD7, MUC1, EGFR, CD20, PD-L1, EIIIB, CD105, or MCMA [128].

**Figure 5:**
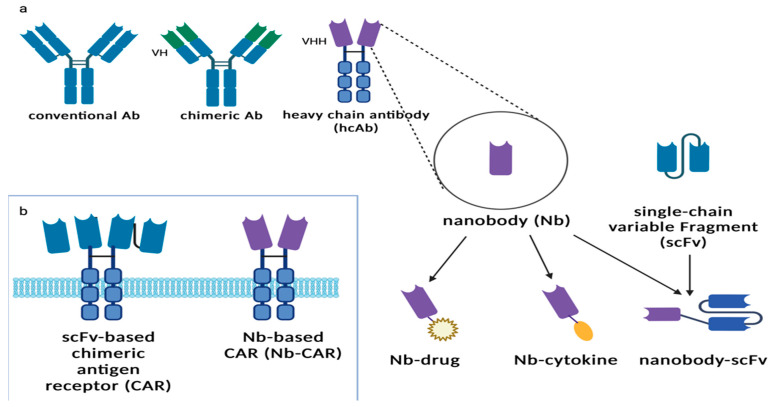
Comparison of conventional and nanobody-based chimeric antigen receptors. (**a**) Schematic diagrams of a conventional antibody (Ab), camelid heavy chain antibody (hcAb), nanobody (Nb) and chimeric antibody, a single chain variable fragment (scFv), and nanobody-scFv. (**b**) Schematic diagrams of conventional scFv-based chimeric antigen receptor (CAR) and Nb-based chimeric antigen receptor (Nb-CAR) [129].

Conventional and nanobody-based chimeric antigen receptors are presented in the Figure 5 [129].

Two trials have analyzed the effectiveness of the preclinical application of Nb-CAR-NK cell therapy [129,130]. To overcome T-cell-originating malignancies, CD7-directed CAR-NK92MI constructs were created. They used their multimers and directly compared monovalent and bivalent Nb-CAR-NK cells. Doubled dCD7Nb.CAR-NK92MI therapy demonstrated superior cytotoxicity against T cell lines and xenograft mouse models of primary T cell tumors over the monovalent CD7Nb [130]. Another approach is based on the incorporation of human CD38-directed nanobodies into a CAR particle. Hambach et al. used a CD38-specific Nb-CARs manufactured from NK-92 cells, which were effective against CD38-expressing multiple myeloma (MM) cell lines and primary patient-derived MM bone marrow samples [129].

## 11. CAR Exosomes in Cancer Therapy as a Novel Anti-Cancer Strategy

**Figure 6:**
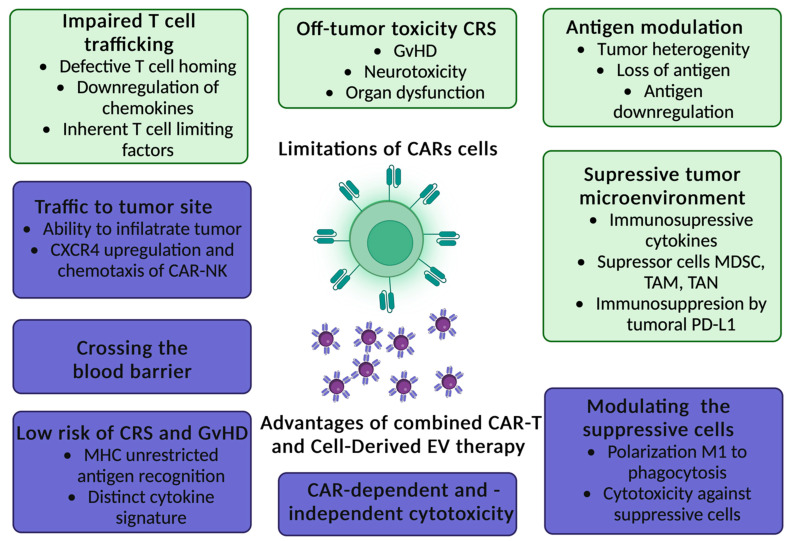
Advantages of combined CARs and Cell-Derived EVs therapy [58,141].

**Figure 7:**
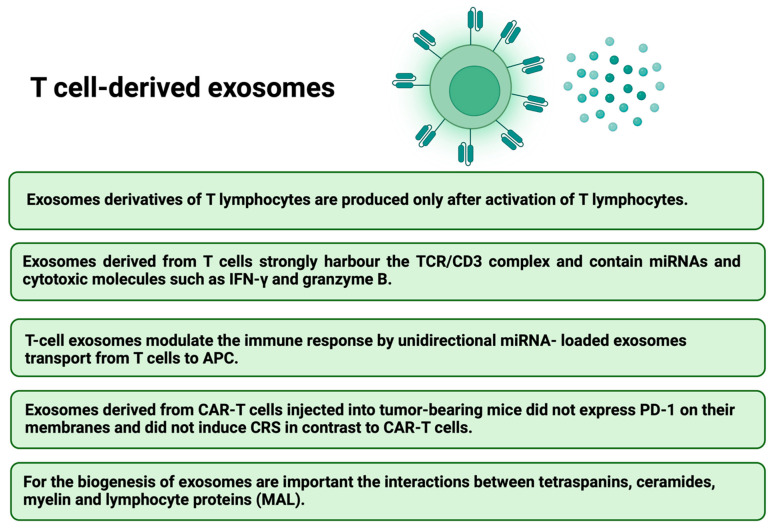
The features of T-cell derived exosomes [142,143,144,145,146].

**Figure 8:**
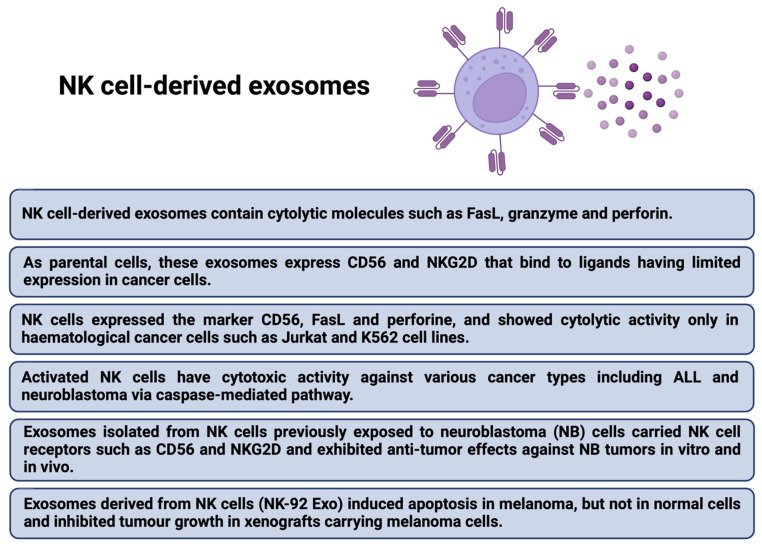
The features of NK-cell derived exosomes [147,148,149,150,151].

The next issue that has begun to receive increasing attention in the context of improving the CARs therapy are exosomes. They are small extracellular vesicles (EVs) secreted by most cell types which play an essential role in intercellular communications and many physiological processes by altering gene regulatory networks or epigenetic recombination [131]. Exosomes derived from effector CAR-T cells have potent anti-tumor effects in treating hematological and non-hematological malignancies [132]. In contrast, tumor-derived exosomes are involved in cancer development, angiogenesis, metastasis, and progression [133,134]. Dendritic-cell-derived exosomes can prime naïve T cells and activate NK cells to reduce the tumor [135]. CAR-T cells release exosomes that carry CAR on their surface, expressing cytotoxic and inhibitory molecules for tumor growth. It was shown, that EVs do not express Programmed cell Death protein 1 (PD1). Therefore, anti-tumor effect of CAR exosomes cannot be weakened by recombinant PD-L1 treatment [136]. Lethal, chemical compounds, such as granzymes and perforins, are delivered to targeted tumor cells by exosomes derived from cytotoxic T lymphocyte (CTL) with CTL surface membrane molecules (CD3, CD8 and TCR). T-cell receptor (TCR) activation boosts the production of CTL-derived exosomes [137]. Additionally, exosomes derived from CAR-T cells may exhibit excellent potential in immunotherapy for drug delivery [138,139]. The administration of CAR exosomes is relatively safe and may be useful in future therapeutic approaches against tumors [132]. Anti-tumor potential of EVs derived from cytokine-stimulated immune cells or EVs engineering has been confirmed [140]. Therefore, the combined CARs and cell-derived EV therapy could bring even better results, the main advantages are presented in Figure 6 [58,141]. Furthermore, both T-cell derived exosomes (Figure 7) [142,143,144,145,146] and NK-cell derived exosomes (Figure 8) [147,148,149,150,151] have their own unique characteristics–different receptors and expression of various molecules.

The bone marrow (BM) microenvironment in hematological malignancies (HMs) comprises heterogeneous populations of nonneoplastic and neoplastic cells, such as hematopoietic stem cells (HSCs), mesenchymal stromal/stem cells (MSCs), and cancer stem cells (CSCs). MSCs actively support hematopoiesis, HSCs can reconstitute the entire hematopoietic system, but CSCs are the HMs initiators and are associated with neoplastic growth and drug resistance. Malignant EVs can interfere with antineoplastic immunity and participate in resistance to treatment by modifying the BM environment in favor of neoplastic cells at the expense of normal HSCs [152]. It was shown that CAR-T EVs may preserve CAR-T cells activity and provide a novel approach to immunotherapy that may be effective against not only hematological malignancies but also solid tumors [153]. Comparison of CAR-T cells and CAR-T cell-derived exosomes is presented in the Table 5 [141].

## 12. Comparison to Other Immunotherapies

To avoid the immune response and establish a microenvironment that permits tumor growth, tumor cells use checkpoint protein signaling [154]. As a pivotal immune checkpoint, the programmed cell death-1 receptor (PD-1, CD279) and cytotoxic T lymphocyte antigen-4 (CTLA-4) are recognized. The treatment of multiple types of advanced solid tumors by preventing molecule-triggered exhaustion is immune checkpoint blockade by anti-PD-1 (pembrolizumab, nivolumab, cemiplimab), PD-L1 (atezolizumab, avelumab, and durvalumab), and anti-CTLA-4 (ipilimumab, tremelimumab) antibodies [155]. CAR-T cells administration in combination with immune checkpoint blockade inhibitors may increase the efficacy of therapy against poorly responding tumors [156]. The tumor microenvironment (TME), combined with physical barriers, makes it difficult to use this method to treat solid tumors. The immunosuppressive microenvironment successfully limits the penetration and mobility of CAR-T cells. In addition to the inhibitors secreted in TME, there are also myeloid-derived suppressor cells (MDSCs), tumor-associated macrophages (TAMs), and regulatory T cells (Tregs) that may weaken the effect of the therapy [157]. Despite all of this, one of the main causes of unresponsive therapy to CARs is poor T cell expansion and short-term T cell persistence. The technical problem preventing CAR-T use is insufficient autologous T cells to achieve clinically relevant doses of CAR-T cells in heavily pretreated patients.

The most effective cancer immunotherapy is T-biAbs-mediated cytotoxicity by recruiting and activating T cells. T-biAbs use an anti-CD3 antibody fragment as the T-cell engaging arm, and the other arm targets tumor cell surface receptors [158]. CD3 × CD19 T-biAb blinatumomab (Blincyto; Amgen, Inc.) was approved by the FDA and EMA for the treatment of adults and children with refractory or relapsed (R/R) pre-B cell acute withlymphoblastic leukemia (pre-B-ALL) [159], and approved for the treatment of adults and children with pre-B ALL in first or second complete remission with minimal residual disease [160,161]. It was shown that bispecific T-cell-engaging antibodies are very effective in anti-cancer therapy [162,163].

Inotuzumab ozogamicin (Besponsa^®^; Pfizer) as a CD22-targeting antibody-drug conjugate (ADC) is approved for adult R/R pre-B ALL, and for the treatment of patients with relapsed or refractory ALL, a group that otherwise has a poor prognosis with standard chemotherapy [164].

The most significant treatment-related toxicity in CAR-T therapy is CRS, caused by immune activation induced by CAR-T cells with fever which can progress to life-threatening capillary leak with hypotension and hypoxia [165]. For the rapid resolution of CAR-T-induced severe CRS tocilizumab is used as an IL-6 receptor antagonist, which has been approved by the FDA and adopted by most clinical trial programs [166,167].

## 13. Conclusions and Future Perspectives

The most promising approaches to management of cancer is personalized immuno-oncology with therapeutic strategies precisely tailored to each patient’s requirements [168]. With advances in modern biotechnology, it is possible to create CAR-T cells with huge potential to treat cancer by combining the exquisite antigen specificity, polyfunctionality, and potency of cellular immunity [169]. Furthermore, CAR constructs such as cytokine-secreting CARs targeted gene delivery into the T cell receptor α constant (TRAC) locus, which shows promise for future clinical use [170]. Despite the success of CAR-T therapy, the key factor for treatment failure is severe life-threatening toxicities, antigen escape, modest anti-tumor activity, and limited tumor infiltration [171]. To avoid these adverse effects, further developments need to be considered to enhance the effectiveness of the adoptive cellular immunotherapy [172]. The main advantage of CAR-NK cells compared to CAR-T is their independence from MHC receptors, so they can be administered without requiring full HLA matching. They can identify malignant or virally infected cells, which often lower their MHC receptors on the surface [173]. To make CAR-NK therapy more standard and available, we should find abundant and effective sources of NK cells. Combining them with other advancements, such as nanobodies-based CARs or exosomes may bring better outcomes. Additionally, a promising approach to enhancing the effectiveness of adoptive cellular immunotherapy is overcoming terminal differentiation or senescence and exhaustion of T cells [174]. In this case, EVs derived from immune cells in combination therapy with drugs may be considered in the treatment of cancer patients, especially effector T and NK cells-derived exosomes with the cytotoxic activity of their original cells [140]. It has been shown that the use of CAR-T cell therapy in diffusing large B-cell lymphoma (DLBCL) has yielded disease control in up to 50% of cases [44]. An important question is what the efficacy of these modern therapies looks like in longer follow-ups.

## Figures and Tables

**Table 1:** CAR-T-cell therapy approved by FDA [43,44,45,46,47,48,49].

Generic Name	Brand Name	Target Antigen	Targeted Disease	Patient Population
Tisagenlecleucel	Kymriah	CD19	B-cell acute lymphoblastic leukemia (ALL)	Children and young adults with refractory or relapsed B-cell ALL
			B-cell non-Hodgkin lymphoma (NHL)	Adults with relapsed or refractory B-cell NHL
Axicabtagene ciloleucel	Yescarta	CD19	B-cell non-Hodgkin lymphoma (NHL)	Adults with relapsed or refractory B-cell NHL
			Follicular lymphoma	Adults with relapsed or refractory follicular lymphoma
Brexucabtagene autoleucel	Tecartus	CD19	B-cell acute lymphoblastic leukemia (ALL)	Adults with refractory or relapsed B-cell ALL
			Mantle cell lymphoma (MCL)	Adults with refractory or relapsed MCL
Lisocabtagene maraleucel	Breyanzi	CD19	B-cell non-Hodgkin lymphoma (NHL)	Adults with relapsed or refractory B-cell NHL
Ciltabtagene autoleucel	Carvykti	BCMA	Multiple myeloma	Adults with relapsed or refractory multiple myeloma
Idecabtagene vicleucel	Abecma	BCMA	Multiple myeloma	Adults with relapsed or refractory multiple myeloma

**Table 2:** Comparison between autologous and allogeneic cells used in CAR-T therapy [55,56,57,58].

	CAR-T Cells	
Sources	Different mixes of helper CD4+ and cytotoxic CD8+ cells T cells.	
	Autologous T cells	Allogeneic T cells
Quality of source	Limitations of quality and quantity T cell number. Various donors of T cells.	Multiple T cell sources from many healthy donors (PB or UCB). Standardized source of cell.
Manufacture	Risk of manufacture in a group for heavily pretreated patients. The limited potency of the CAR-T cellular product is because the patient’s T lymphocytes treated with chemotherapy are more differentiated with lower proliferation capacity and rapid exhaustion.	The starting material is high quality from a healthy donor.
Risk of contamination	Risk of contamination with cancer cells in patient blood.	Minimal risks of cancer cell contamination, source form healthy donor blood.
Persistence	Increased in vivo persistence compared with allogeneic CAR-T cells due to lack of immune rejection from the host.	Decreased in vivo persistence due to higher immunogenicity.
Risk of GVHD	Low	High
Scalability	Low-personalized product for one patient	High—one product for many patients
Acute side effects	It may cause GVHD, CRS and neurotoxicity.	Barely cause GVHD, may even protect against GVHD. Lack of CRS and neurotoxicity.

**Table 3:** Cytotoxic mechanism of CAR-NK and CAR-T cells.

Mechanism	CAR-NK Cells	CAR-T Cells
Chimeric antigen receptor	CARs cells can aim for specific tumor antigen	
Antigen presentation	NK cells can specifically recognize the cells that lack the expression of self-MHC class I molecules [65]. Enhancing the antigen presentation to T cells by killing the immature DC while promoting the IFN g and TNF-a mediated maturation of DC [66].	They can recognize antigens regardless of MHC presentation. However, they are limited to the recognition of structures expressed at the surface [28,29].
Transduction efficiency	lower	higher
In vivo persistence	worse	better
Fas/FasL	The Fas-FasL is a major apoptosis pathway via caspase-dependent activation. The antigen-negative cancer cells can be targeted via FAS and Fas L axis, independent of presenting death receptors by the cancer cell. It is estimated that the functions of this pathway may be pivotal in the heterogeneous environment of the tumor [38,39].	
Cytolytic granules	CARs cells lyse the antigen-positive cancer cells mainly by the cytolytic granules. The perforins are inducing pore formation in the cancer membrane, forming the access for granzymes. In the cytoplasm, they could induce apoptotic cell death in a caspase-dependent or independent way. Therefore, cytolytic degranulation is assumed to be the most important mechanism of cell killing by CAR-T cells [6]. Cytokine production induces cell death via secondary mechanisms, such as enhancing CARs, Fas, or TRAIL pathways. They trigger several anti-tumor immune responses, including the enhancement of the cytotoxic response, recruitment, and activation of innate immune cells [67].	
	There were attempts to use ectopically expressed chimeric granzyme B. This approach could enhance NK-cell degranulation and efficient producing cytolytic granules [68,69].	Cytolytic granules secretion by CAR-T cells mediates tumor lysis via upregulating IFN-gamma on stromal cells [41]. That leads to immune cell modulations, such as the polarization of macrophages to the antitumoral M1 phenotype [42].
Checkpoint inhibitors	Prevention of the interaction of inhibitory receptors with their respective ligands leads to inhibition of NK cell suppression [70]. Additionally, checkpoint molecules can enable tumor escape from NK cell vigilance [71].	CAR-T cells can secrete immune checkpoint inhibitors to overcome immunosuppression of tumors (e.g., anti-PD-1/PD-L1/CTLA-4) for enhanced strength, effectiveness, and persistence of CAR-T therapy [72,73,74,75,76].

**Table 4:** Advantages and disadvantages of CAR-T [58] and CAR-NK cells [105].

CAR-T Cells		CAR-NK Cells	
Advantages	Disadvantages	Advantages	Disadvantages
More extended experience of the centers in working on the process of developing and delivering CAR-T cells. Easier freezing and storage. High rates of circulating T-lymphocytes. Strong cytotoxicity. Efficient results in hematologic cancers.	When not modified, only for autologous use. The high risk for cytokine release syndrome, neurotoxicity, or graft-versus-host-disease. Various ratios of CD8+ and CD4+ in different donors.	Allogenic or haploidentical NK cells can be used. High feasibility for manufacturing of ‘’Off-the-shelf’’ product. The low risk for cytokine release syndrome, neurotoxicity, and graft-versus-host disease. Triggering the ADCC dependent cytotoxicity. Various activating and inhibitory receptors	Great sensitivity to freezing and thawing. Low rates of circulating NK cells. Cytokine support is crucial for their persistence. Minimal worldwide experience in manufacturing. Dysfunction and exhaustion through suppressive cells such as Tregs and suppressive cytokines.

**Table 5:** Comparison of CAR-T cells and CAR-T cell-derived exosomes [141].

Event	CAR-T Cells	CAR-T Cell-Derived Exosomes
Cross the blood barrier	−	++
Cytokine releasing syndrome	++	−
Neurotoxicity and GvHD	++	−
Reprograming and act against suppressive cells	−	++
Efficiency against solid tumors	+	++

## Data Availability

Not applicable.
